# Supplementary figures and images for: KLF4 is required for suppression of histamine synthesis by polyamines during bone marrow-derived mast cell differentiation
Source: PLoS One. 2020 Feb 26;15(2):e0229744. doi: 10.1371/journal.pone.0229744 (PMC7043748; doi:10.1371/journal.pone.0229744)

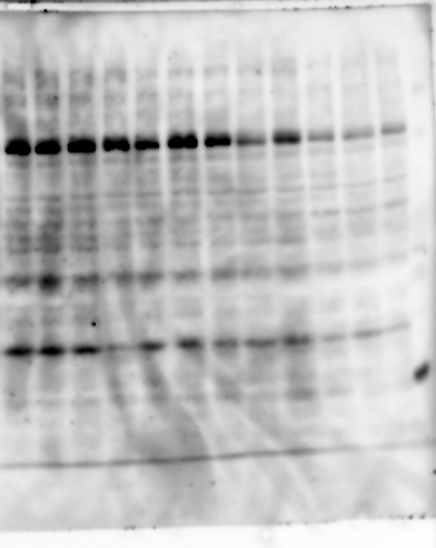

Supplement: S1 Fig — (PDF) [file pone.0229744.s001.pdf]

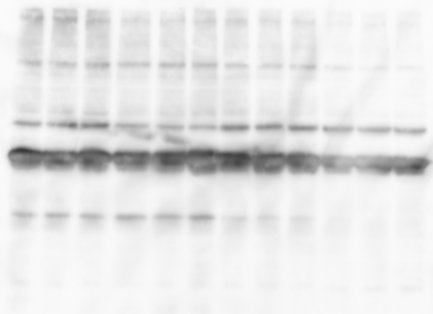

Supplement: S2 Fig — (PDF) [file pone.0229744.s002.pdf]
