# Supplementary material for: KLF4 is required for suppression of histamine synthesis by polyamines during bone marrow-derived mast cell differentiation
Source: PLoS One. 2020 Feb 26;15(2):e0229744. doi: 10.1371/journal.pone.0229744 (PMC7043748; doi:10.1371/journal.pone.0229744)
Supplement: S3 Fig — (PDF) [file pone.0229744.s003.pdf]

Supporting Information

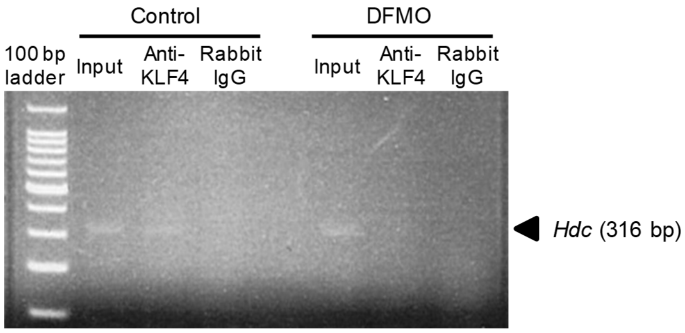

Fig S3. Chromatin immunoprecipitation assay during BMMC differentiation in the absence or presence of DFMO. Cells (5 x 10^6 cells) on day 14 during BMMC differentiation fixed with 1% formaldehyde and used for ChIP assay. ChIP assays were performed using Simple ChIP® Enzymatic IP Kit (Cell signaling) following manufacture's protocol. Antibody, GKLF (H-180) (sc-20691; Santa Cruz) were used for immunoprecipitation. PCR was performed using the specific primer pairs of *Hdc* promoter; forward, 5′-TGG CAA TTC TTC CCC CTT ACG-3′, reverse, 5′-GCT CCT GCC CTG GCT TCT CTA T-3′.
