# Supplementary material for: KLF4 is required for suppression of histamine synthesis by polyamines during bone marrow-derived mast cell differentiation
Source: PLoS One. 2020 Feb 26;15(2):e0229744. doi: 10.1371/journal.pone.0229744 (PMC7043748; doi:10.1371/journal.pone.0229744)
Supplement: S4 Fig — (PDF) [file pone.0229744.s004.pdf]

Supporting Information

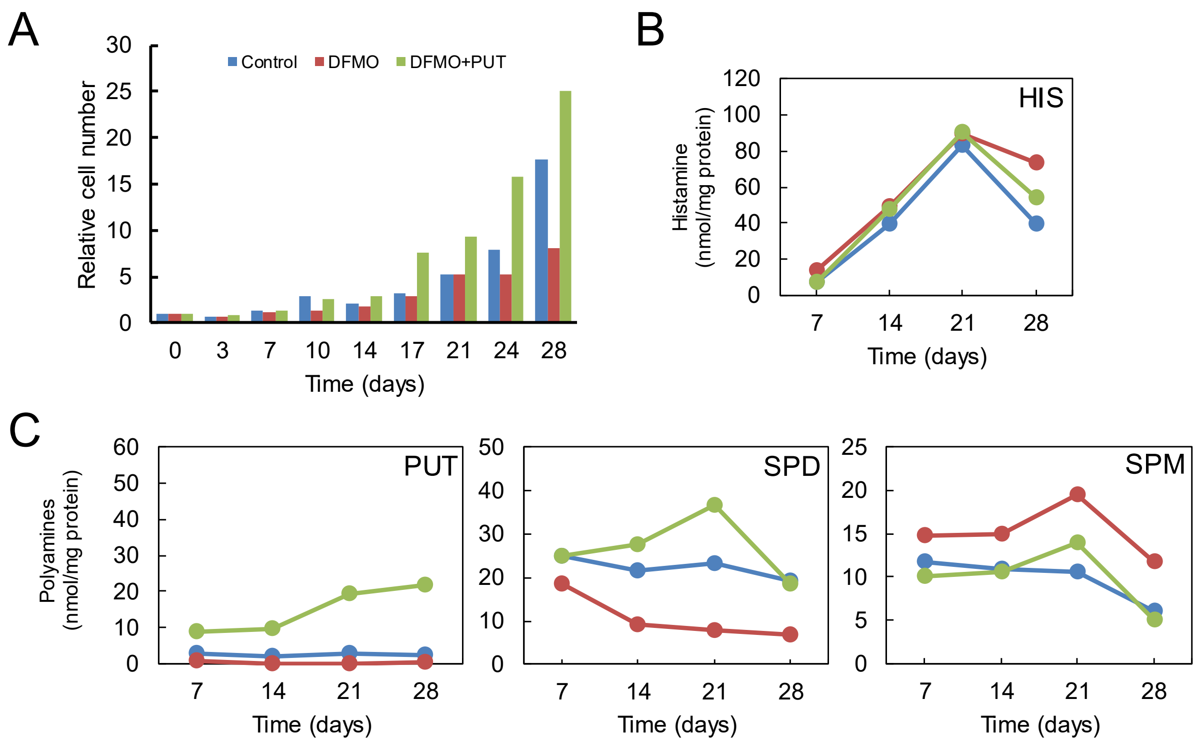

Fig S4. Cell number and intracellular amines in BMMCs in the absence or presence of DFMO and putrescine. (A) Cells were cultured with 0.1 mM DFMO and 0.1 mM putrescine (PUT) treatment from day 3. The relative cell number on day 0 is shown as 1, n = 2 duplicates. During BMMC differentiation, cells on day 7, 14, 21, and 28 were used for analysis of intracellular histamine (B) and polyamines (C) by HPLC. Mean was shown, n = 2 duplicates.
